# Updated Strategies in Non-Culprit Stenosis Management of Multivessel Coronary Disease—A Contemporary Review

**DOI:** 10.3390/medicina60020263

**Published:** 2024-02-02

**Authors:** Rares-Dumitru Manuca, Alexandra Maria Covic, Crischentian Brinza, Mariana Floria, Cristian Statescu, Adrian Covic, Alexandru Burlacu

**Affiliations:** 1 Institute of Cardiovascular Diseases “Prof. Dr. George I.M. Georgescu”, 700503 Iasi, Romania; rares.manuca@d.umfiasi.ro (R.-D.M.); covic_maria-alexandra@d.umfiasi.ro (A.M.C.); cristian.statescu@umfiasi.ro (C.S.); 2 Faculty of Medicine, University of Medicine and Pharmacy “Grigore T Popa”, 700115 Iasi, Romania; floria.mariana@umfiasi.ro (M.F.); adrian.covic@umfiasi.ro (A.C.); 3 Internal Medicine Clinic, “St. Spiridon” County Clinical Emergency Hospital Iasi, 700111 Iasi, Romania; 4 Nephrology Clinic, Dialysis, and Renal Transplant Center, “C.I. Parhon” University Hospital, 700503 Iasi, Romania

**Keywords:** multivessel coronary artery disease, acute coronary syndrome, revascularization strategies, complete revascularization, percutaneous coronary intervention

## Abstract

The prevalence of multivessel coronary artery disease (CAD) in acute coronary syndrome (ACS) patients underscores the need for optimal revascularization strategies. The ongoing debate surrounding percutaneous coronary intervention (PCI), coronary artery bypass grafting (CABG), hybrid interventions, or medical-only management adds complexity to decision-making, particularly in specific angiographic scenarios. The article critically reviews existing literature, providing evidence-based perspectives on non-culprit lesion revascularization in ACS. Emphasis is placed on nuances such as the selection of revascularization methods, optimal timing for interventions, and the importance of achieving completeness in revascularization. The debate between culprit-only revascularization and complete revascularization is explored in detail, focusing on ST-elevation myocardial infarction (STEMI) and non-ST-elevation myocardial infarction (NSTEMI), including patients with cardiogenic shock. Myocardial revascularization guidelines and recent clinical trials support complete revascularization strategies, either during the index primary PCI or within a short timeframe following the culprit lesion PCI (in both STEMI and NSTEMI). The article also addresses the complexities of decision-making in NSTEMI patients with multivessel CAD, advocating for immediate multivessel PCI unless complex coronary lesions require a staged revascularization approach. Finally, the article provided contemporary data on chronic total occlusion revascularization in ACS patients, highlighting the prognostic impact. In conclusion, the article addresses the evolving challenges of managing multivessel CAD in ACS patients, enhancing thoughtful integration into the clinical practice of recent data. We provided evidence-based, individualized approaches to optimize short- and long-term outcomes. The ongoing refinement of clinical and interventional strategies for non-culprit lesion management remains dynamic, necessitating careful consideration of patient characteristics, coronary stenosis complexity, and clinical context.

## 1. Introduction

Multivessel coronary artery disease (CAD) is a common occurrence in at least half of patients with acute coronary syndrome (ACS), raising significant concerns regarding the optimal management of non-culprit lesions [1,2]. Myocardial revascularization strategies (techniques and timing) regarding non-culprit vessels (defined as non-infarct-related arteries) were extensively discussed in the literature [1]. Nevertheless, new reports were disseminated during the 2023 European Society of Cardiology (ESC) congress, demanding thoughtful integration into clinical practice to improve the outcomes of ACS patients [2].

Although the main clinical trials discussed during the 2023 ESC congress may not introduce revolutionary changes in multivessel CAD management in ACS patients, their significance lies in the robustness of the evidence they contribute to the ongoing debate [2,3]. Thus, while not revolutionary, these studies offer substantial input and require careful consideration for integration into the current state of practice. These studies, by design and scale, provide valuable real-world insights that can help refine clinical decision-making [2,3].

However, the best optimal approach for myocardial revascularization in the case of multivessel disease remains a subject of ongoing investigation [4,5]. Percutaneous coronary intervention (PCI), coronary artery bypass grafting (CABG), hybrid interventions, or medical-only management constitute viable treatment strategies in multivessel ACS patients. The different impact on clinical outcomes of these revascularization strategies has led to ongoing debates and a need for more straightforward recommendations, especially in specific angiographic scenarios [4,5].

In a registry encompassing 16,320 patients diagnosed with CAD who underwent coronary angiography, 58.3% of the cohort had multivessel disease, defined by at least 50% stenosis in more than one coronary artery. Notably, individuals with multivessel CAD tended to be older. They exhibited a higher prevalence of comorbidities and risk factors, including arterial hypertension, dyslipidemia, diabetes mellitus, a history of myocardial infarction, and chronic kidney disease (CKD) [6]. In another registry with consecutive ACS patients (n = 680), 47.8% of individuals aged 50 years or younger exhibited two- or three-vessel CAD. Also, older patients (51–65 years) had a high prevalence of multivessel CAD, documented in 62.8% of cases [2]. Furthermore, multivessel disease was significantly more prevalent in ACS patients with cardiogenic shock as compared to those without cardiogenic shock (59% vs. 46%, *p* < 0.001) [7]. These findings underscore the need to evaluate the optimal subsequent revascularization strategy in ACS patients.

Multivessel CAD in patients with ACS may also harm short- and long-term major clinical outcomes. In a multivariate analysis including ACS patients with cardiogenic shock, multivessel CAD was associated with a significantly higher risk of mortality during follow-up (HR 1.22, 95% CI, 1.06–1.39, *p* < 0.01) [7]. In a cohort of 1342 patients with non-ST-elevation myocardial infarction (NSTEMI) and multivessel CAD, all-cause mortality at 12 months varied widely according to the treatment strategy [8]. Higher mortality (25.3%) was observed in the medical-only treatment group, compared to the 11.7% mortality rate in the PCI group and 11.1% in the CABG group [8].

Consequently, we aimed to critically review the existing literature on non-culprit lesion management in the setting of ACS patients. Our manuscript serves as a comprehensive resource, providing nuanced (evidence-based) perspectives on non-culprit lesion revascularization in ACS, examining revascularization methods selection, optimal timing for interventions, and the critical consideration of achieving completeness in revascularization. Thus, the review provides valuable insights that contribute to refining clinical and interventional strategies for non-culprit lesion management in ACS patients. While acknowledging the unblinded status of patients regarding their multivessel disease as a source of bias, we employed a comprehensive approach, evaluating a spectrum of clinical outcomes, including unplanned myocardial revascularization, recurrent myocardial infarction, and mortality. 

## 2. Complete versus Culprit-Only Revascularization: A Cornerstone of Acute Coronary Syndrome Management

The choice between culprit-only and complete revascularization in the context of myocardial infarction has been the subject of debate within the cardiovascular community [4]. To provide a comprehensive assessment, it is crucial to evaluate the long-term prognosis and clinical outcomes associated with each approach [4].

### 2.1. Decision-Making in STEMI with Multivessel CAD

In patients with ST-elevation myocardial infarction (STEMI) and multivessel CAD, the most appropriate revascularization strategy is determined through a Heart Team consensus approach. The available revascularization options include (a) multivessel PCI during the index primary PCI, (b) PCI of the infarct-related artery (IRA) only, followed by staged PCI of non-IRA arteries, (c) PCI of the IRA only with an ischemia-guided approach for treating non-IRA coronary stenosis, or (d) PCI of the IRA only with elective CABG in selected patients.

#### Guidelines and Clinical Trials

The 2021 American College of Cardiology and the American Heart Association (ACC/AHA) guidelines for coronary artery revascularization recommended staged PCI post-successful primary PCI in selected hemodynamically stable patients (class I recommendation, Level of Evidence A) [9]. Also, in selected patients with low complexity multivessel CAD, ACC/AHA guidelines advocated for PCI of non-IRA during the index primary PCI to optimize long-term cardiovascular outcomes (class II recommendation, Level of Evidence b). Ideal candidates for non-IRA revascularization include those with a large area of viable myocardium at risk and minimal comorbidities that could increase the risk of percutaneous revascularization procedures [9].

These recommendations were supported by the COMPLETE trial (Complete versus Culprit-Only Revascularization Strategies to Treat Multivessel Disease after Early PCI for STEMI), which enrolled 4041 patients with long-term follow-up (median three years) [10]. The study demonstrated that staged PCI of non-IRA (performed within 45 days of the index PCI) was superior to culprit lesion-only PCI in reducing the composite risk of cardiovascular death or myocardial infarction (HR 0.74, 95% CI, 0.60–0.91, *p* = 0.004). Moreover, the composite risk of cardiovascular death, myocardial infarction, or ischemia-driven revascularization was significantly lower in the complete revascularization arm (HR 0.51, 95% CI, 0.43–0.61, *p* < 0.001). Conversely, elective CABG remains a viable alternative in STEMI cases with complex multivessel CAD, given the exclusion of planned surgical revascularization patients from this study [10].

The CvLPRIT trial (Complete versus Lesion-only Primary PCI) analyzed 296 patients from the United Kingdom who were randomized to either complete revascularization during the index hospitalization (n = 150) or IRA-only PCI (n = 146) [11]. The primary composite outcome included all-cause mortality, recurrent myocardial infarction, heart failure, and ischemia-driven revascularization during 12 months of follow-up. Among those who underwent complete revascularization, 10.0% experienced the primary endpoint, compared to 21.2% of patients from the IRA-only PCI arm (HR 0.45; 95% CI, 0.24–0.84, *p* = 0.009). Other outcomes were similar in both treatment groups, including major bleedings, contrast-induced nephropathy, stroke, and ischemic burden (on myocardial perfusion scintigraphy). Therefore, achieving complete myocardial revascularization during the index hospitalization is reasonable to reduce the 12-month risk of adverse events in patients who underwent primary PCI [11].

The 2023 ESC guidelines on the management of ACS have also endorsed complete myocardial revascularization, either during the index primary PCI or within 45 days (class Ia recommendation), emphasizing the importance of angiographic coronary stenosis severity, clinical status, and comorbidities in decision-making [4].

The ongoing debate between culprit-only revascularization and complete myocardial revascularization in patients with STEMI underscores the intricate nature of decision-making in CAD management (Table 1) [9,12]. Both ACC/AHA and ESC guidelines acknowledged the benefit of complete myocardial revascularization on short- and long-term outcomes in ACS patients. Thus, complete myocardial revascularization (PCI or CABG) should be a cornerstone of ACS management to achieve optimal patient outcomes [4,12].

### 2.2. Decision-Making in NSTEMI with Multivessel CAD

The approach to myocardial revascularization in NSTEMI patients is a complex decision that involves weighing multiple factors [13]. The goal is to tailor the treatment to the individual patient’s needs, balancing the potential benefits of more comprehensive revascularization with the risks associated with more extensive procedures (multivessel stenting). Decisions should be made collaboratively, often in a heart team setting (involving the patient in decision-making) [13,14].

A meta-analysis published in 2015 included six observational studies on patients presenting with NSTEMI and multivessel CAD (no randomized clinical trial was available) [15]. Multivessel PCI was associated with a similar risk of mortality and myocardial infarction as compared to culprit-only PCI (respectively, OR 0.85, 95% CI, 0.70–1.04, *p* = 0.114 and OR 0.75, 95% CI, 0.43–1.32, *p* = 0.319). Nevertheless, multivessel PCI was linked to a lower long-term risk of major adverse cardiovascular events and unplanned revascularization, compared to culprit-only PCI (respectively, OR 0.69, 95% CI, 0.51–0.93, *p* = 0.015 and OR 0.64, 95% CI, 0.45–0.93, *p* = 0.018). Therefore, multivessel PCI rather than culprit-only PCI should be considered in hemodynamically stable NSTEMI patients to reduce the long-term risk of major adverse events and unplanned revascularization [15].

Recent data confirmed the benefit of multivessel PCI over culprit-only PCI in patients presenting with NSTEMI [16]. Patients who underwent immediate multivessel PCI had a lower risk of all-cause mortality during long-term follow-up (median 59 months) compared to the culprit-only group (HR 0.592, 95% CI, 0.364–0.960, *p* = 0.034) [16]. Staged multivessel PCI was also associated with a lower incidence of major adverse cardiovascular events and all-cause mortality compared to culprit-only PCI (*p* < 0.001 for both). Moreover, patients with more complex coronary lesions (SYNTAX score > 22) had a more significant reduction in major adverse cardiovascular events when multivessel PCI was performed as a staged procedure rather than in an immediate setting during the index PCI [16]. In another large observational study, immediate multivessel PCI was associated with a lower mortality risk during 4.1 years of follow-up than culprit-only PCI (*p* = 0.0005) [17]. Notably, multivessel PCI could improve in-hospital mortality in patients with NSTEMI and cardiogenic shock, but these data should be confirmed in large clinical trials [18].

Thus, it is reasonable to perform immediate multivessel PCI in patients with NSTEMI unless they exhibit complex coronary lesions when a staged revascularization strategy should be adopted [16,17].

## 3. Optimizing Outcomes in Acute Coronary Syndromes: Immediate versus Staged Non-Culprit PCI

While clinical trials consistently reported the benefit of complete myocardial revascularization, appropriate timing of PCI (or CABG) of non-culprit stenosis should be established to achieve optimal long-term outcomes without increasing the risk of short-term adverse events (Table 2) [4,10].

In a recent study, the MULTISTARS AMI trial, presented during the 2023 ESC congress, 840 STEMI patients (without cardiogenic shock) with multivessel CAD were randomized to immediate multivessel PCI or staged multivessel PCI (19–45 days after culprit lesion PCI) [2,19]. The primary composite outcome (all-cause mortality, non-fatal myocardial infarction, unplanned revascularization, stroke, or hospitalization for heart failure) at one-year follow-up had a lower incidence in patients who underwent immediate multivessel PCI as compared to staged PCI (respectively, 8.5% and 16.3%; RR 0.52, 95% CI, 0.38–0.72, *p* < 0.001). Notably, the severity of non-culprit coronary lesions was estimated visually (defined as at least 70% stenosis) [2]. Nevertheless, the results cannot be extrapolated to all STEMI patients due to several exclusion criteria: required CABG or hybrid revascularization (or other cardiac surgery), left main stenosis, chronic total occlusion (CTO), or kidney disease (estimated glomerular filtration rate <30 mL/min/1.73 m^2^) [2,19]. Therefore, it is reasonable to apply an immediate revascularization strategy in hemodynamically stable STEMI patients and multivessel disease (all amendable to PCI) without left main disease, not requiring emergency cardiac surgery (including due to mechanical complications) or hybrid revascularization [2,19].

Similar results were reported in another recent randomized clinical trial (BIOVASC) that included 764 patients with STEMI or NSTEMI and multivessel CAD [3]. Patients were divided into two arms: an immediate complete revascularization group and a staged revascularization group (within six months following the index primary PCI) [3]. The primary composite outcome incidence (all-cause death, myocardial infarction, unplanned revascularization, or cerebrovascular events) was similar in both groups (HR 0.78, 95% CI, 0.55–1.11). Myocardial infarction and unplanned revascularization occurred less frequently in the immediate revascularization group (respectively, HR 0.41, 95% CI, 0.22–0.76, *p* = 0.0045, and HR 0.61, 95% CI, 0.39–0.95, *p* = 0.030) [3]. Another study confirmed the results, favoring immediate complete revascularization [20].

In a post-hoc analysis of a randomized clinical trial (FLOWER-MI), 1-year primary composite outcome risk (all-cause mortality, non-fatal myocardial infarction, and unplanned revascularization) was similar in immediate and staged non-IRA PCI (in the first five days following the index primary PCI) [21]. However, only 3.8% of patients underwent immediate multivessel PCI [21]. In a randomized clinical trial (discontinued prematurely), major adverse cardiovascular events at one year had a similar incidence in both treatment groups, immediate and staged multivessel PCI (HR 1.60, 95% CI, 0.65–3.91) [22]. Nevertheless, some studies reported a potential increased risk of adverse events in immediate non-culprit stenosis PCI compared to staged PCI, warranting further investigation [23,24].

Although a recent clinical trial supports the immediate multivessel PCI (with improved or at least non-inferior outcomes to staged PCI), one study has highlighted the potential for overestimating non-culprit coronary stenosis during primary PCI [25]. Several mechanisms could be involved, including inflammation, prothrombotic state, microvascular dysfunction, and increased sympathetic activity [25]. Notably, 13.3% of non-culprit stenoses initially assessed as greater than 70% during the primary PCI were found to have reduced severity to less than 70% at a secondary evaluation one month later. Therefore, at least 13% of patients could avoid PCI and subsequent stent implantation. However, these results should be confirmed in large randomized clinical trials [25].

## 4. Myocardial Revascularization in Cardiogenic Shock: Balancing Short- and Long-Term Outcomes

Patients with cardiogenic shock (CS) due to ACS pose a considerable challenge for decision-making in terms of medical, interventional, and surgical management [4,26]. This complexity is heightened by the substantial likelihood (almost 80%) of these patients have multivessel CAD. Therefore, optimizing medical, interventional, and surgical strategies is required to improve their outcomes and survival [4,26].

In patients with ACS and CS, ESC guidelines recommend PCI of the culprit artery only, and staged PCI could be considered for non-culprit coronary artery stenosis [4]. Similarly, the ACC/AHA guidelines advocate against routine PCI of the non-culprit artery during primary PCI in patients with STEMI and CS, as it could increase the risk of death and acute kidney injury [12].

These recommendations were based on the results from a randomized clinical trial (CULPRIT-SHOCK) [27]. In this trial, 706 patients with CS and multivessel CAD were divided into two treatment arms: PCI of the culprit lesion only (and staged PCI of non-culprit stenosis) and multivessel PCI at the index procedure [27]. The composite endpoint of mortality or renal replacement therapy at 30 days had a lower incidence in the culprit-only PCI group (RR 0.83, 95% CI, 0.71–0.96, *p* = 0.01). Also, the mortality risk was significantly lower in the culprit-only PCI group (RR 0.84, 95% CI, 0.72–0.98, *p* = 0.03) [27]. Nevertheless, no differences were reported between groups at one year for mortality and recurrent myocardial infarction [28]. However, patients from the culprit-only group had a higher risk of repeated revascularization (RR 3.44, 95% CI, 2.39–4.95) and rehospitalization for heart failure (RR 4.46, 95% CI, 1.53–13.04) during the first year of follow-up [28].

One meta-analysis before the CULPRIT-SHOCK trial reported an increased short-term mortality risk in patients with multivessel PCI compared to culprit-only PCI (RR 1.26, 95% CI, 1.12–1.41, *p* = 0.001) [29]. Notably, another meta-analysis that included the CULPRIT-SHOCK trial reported a similar risk of short-term death in both treatment groups, culprit-only PCI and multivessel PCI (OR 1.14, 95% CI, 0.9–1.43) [30].

Therefore, based on all available data and due to potential heterogeneity across studies in meta-analyses, it is reasonable to perform culprit-only PCI at the index primary PCI to reduce short-term mortality (Table 3) [27]. Nevertheless, complete myocardial revascularization should be achieved early after primary PCI to reduce long-term adverse events, including mortality risk (during the hospital stay or within 45 days as recommended in hemodynamically stable STEMI patients) [4].

Regarding the method of revascularization, PCI versus CABG, one trial published in 2005 documented a similar survival rate at 30 days in patients treated with PCI as compared to CABG group (55.6% vs. 57.4%, *p* = 0.86) [31]. Also, the 1-year survival rate was similar between treatment groups (51.9% in the PCI group vs. 46.8% in the CABG group, *p* = 0.71). However, patients from the CABG group had a higher prevalence of diabetes, multivessel CAD (including three-vessel disease), as well as left main stenosis [31]. The results might differ in contemporary ACS patients treated with a new-generation drug-eluting stent [4].

## 5. Non-Culprit Stenosis Revascularization: Weighing PCI and CABG

The decision of revascularization and the selection of the optimal revascularization strategy for ACS patients with multivessel disease is a complex decision that requires careful consideration of individual patient characteristics, the extent and location of CAD, and individual preferences (Table 4) [4]. A thorough assessment of each revascularization option’s potential benefits and risks is essential to optimize patient outcomes [4].

Revascularization of intermediate non-culprit coronary stenoses (PCI or CABG) may not significantly improve long-term patient outcomes [32]. Careful assessment of the functional status of coronary stenosis and the clinical context is essential before proceeding with myocardial revascularization. In one study, deferring PCI in patients with intermediate coronary stenosis (FFR ≥ 0.75) was associated with a similar rate of death during 15 years of follow-up as compared to routine PCI (RR 1.06, 95% CI, 0.69–1.62, *p* = 0.79). Notably, deferred PCI was linked to a decreased risk of myocardial infarction (RR 0.22, 95% CI, 0.05–0.99, *p* = 0.03) [32]. Nevertheless, in assessing non-culprit lesions in multivessel disease using FFR, it is crucial to acknowledge the potential for overestimation [33,34]. This data highlights the complexity of physiological assessments, emphasizing the importance of integrating both anatomical and physiological considerations in clinical decision-making for ACS patients with multivessel CAD [33,34].

Moreover, patients who underwent CABG could have worse long-term survival as compared to the general population [35]. In one study with 30 years of follow-up, CABG patients exhibited an increased mortality risk (adjusted HR 1.76, 95% CI, 1.62–1.91) compared to the general population [35]. Moreover, patients who underwent CABG had a 25-fold increased risk of cardiac death (from myocardial infarction) in the first month after the surgery. Therefore, adherence to guidelines and revascularization of non-culprit lesions should be performed on a case-by-case basis [35]. 

In an early study published in 1991, 1387 consecutive patients with STEMI who received thrombolytic therapy were enrolled [36]. From the entire cohort, 303 patients underwent CABG before hospital discharge in an emergency or deferred setting (respectively, <24 h and >24 h). CABG was recommended in case of multivessel CAD, failed PCI, left main disease, ventricular pump dysfunction refractory to standard therapy, mitral regurgitation, or ventricular septal rupture [36]. The authors reported a similar rate of in-hospital death in CABG patients as compared to those from the non-surgical arm (7% vs. 6%). Also, long-term mortality was similar in both groups (7% in CABG patients and 6% in the non-surgical group). Notably, left ventricular ejection fraction (LVEF) was improved in patients who underwent CABG (*p* = 0.0360). These results highlight the safety of CABG in patients with acute myocardial infarction, even in those who received thrombolytic therapy. The study identified several scenarios where CABG may be the preferred option for myocardial revascularization (over PCI), including failed PCI, incomplete revascularization, and mechanical complications [36].

The timing of CABG surgery for non-culprit coronary stenosis in ACS patients could significantly affect in-hospital mortality [37]. The highest mortality rate (14.2%) was reported when emergency CABG was performed within the first 6 h [37]. This risk progressively decreased to its lowest point (2.7%) when the surgery was delayed beyond 15 days [37]. These findings suggest that for patients who do not require immediate cardiac intervention (due to mechanical complications of myocardial infarction, failed PCI, or incomplete revascularization), postponing CABG for non-culprit coronary stenosis can significantly reduce the risk of in-hospital mortality. This delay allows time for the patients to stabilize and improve their overall condition before undergoing the surgery [37].

Some data could be extrapolated from studies comparing myocardial revascularization by PCI and CABG in patients with NSTEMI and multivessel CAD [38]. CABG, compared to PCI, was linked to an increased risk of stroke (*p* = 0.03), myocardial infarction (*p* = 0.03), and acute renal injury (*p* < 0.0001), but with a lower risk of unplanned revascularization at one month and one year (respectively, *p* < 0.0001 and *p* < 0.000). The mortality risk was similar in both treatment arms during one year of follow-up (*p* = 0.58) [38]. In another analysis, diabetic patients had a similar risk of myocardial infarction, death, and stroke, irrespective of the revascularization method [5]. However, CABG was associated with a reduced risk of revascularization (*p* = 0.03) but a higher rate of acute kidney injury and bleeding (*p* < 0.0001 for both) [5]. Acknowledging the current limitations in randomized controlled trials on staged PCI versus staged CABG and the limited scientific support for the hybrid approach, further research is essential to enhance the understanding of optimal revascularization strategies in ACS patients with multivessel CAD.

## 6. Chronic Total Occlusion Revascularization in Acute Coronary Syndrome: A Quest for Optimal Strategies

The coexistence of CTO in a non-culprit coronary artery could negatively influence the outcomes of patients presenting with myocardial infarction [39]. The emphasis of ESC guidelines on ACS management is focused on achieving complete myocardial revascularization [4]. The selection between PCI, CABG, and hybrid revascularization strategies should be based on clinical status, co-morbidities, and the complexity of coronary stenosis [4]. This approach acknowledges the nuanced interplay of clinical factors and lesion complexity in determining the optimal course of revascularization for myocardial infarction patients with concurrent CTO in a non-culprit artery (Table 5).

The need for CTO revascularization resides in the prognostic impact within the context of ACS. In the HORIZONS-AMI clinical trial, 3,283 patients with STEMI who underwent primary PCI were enrolled [39]. Multivessel disease without CTO was documented in 45.0% of patients, while 8.6% had multivessel disease with CTO (in a non-culprit artery). The presence of CTO was associated with a reduced rate of TIMI 3 flow achievement (*p* = 0.0003), myocardial blush (*p* = 0.0002), and ST-segment resolution (*p* = 0.0001). Moreover, patients with non-culprit CTO had almost three-fold higher 30-day mortality risk compared to those without CTO (HR 2.88, 95% CI, 1.41–5.88, *p* = 0.004) and a two-fold higher mortality risk during three years of follow-up (HR 1.98, 95% CI, 1.19–3.29, *p* = 0.009) [39].

Similar results were documented in another study that enrolled 8679 patients with STEMI treated with primary PCI [40]. CTO in a non-culprit artery was observed in 11.6% of patients. Patients with non-culprit CTO had a higher 30-day mortality risk (HR 1.91, 95% CI, 1.54–2.36, *p* < 0.001) and an increased risk of mortality at five years (HR 1.66, 95% CI, 1.42–1.95, *p* < 0.001) [40]. In addition, STEMI patients with non-culprit CTO had a significantly reduced left ventricular ejection fraction, lower myocardial salvage index, and more extensive area of infarction assessed using cardiac magnetic resonance (CMR) [41]. These data were confirmed in a meta-analysis involving a large cohort of patients that documented an increased in-hospital mortality risk in CTO patients (*p* < 0.001), as well as a more extended hospital stay than in non-CTO arm (*p* = 0.001) [42].

Although the aim of complete myocardial revascularization constitutes a standard desiderate, the available literature on the comparative impact of PCI, CABG, or hybrid approaches on the clinical outcomes of ACS patients with CTO is currently limited [43]. Also, data regarding non-culprit PCI efficacy and outcomes improvement is discrepant. In a randomized clinical trial involving 302 patients with STEMI and non-culprit CTO, those who underwent PCI for CTO had a similar rate of major adverse cardiac events compared to patients from non-PCI group during five years (HR 1.03, 95% CI, 0.54–1.98, *p* = 0.93). Notably, cardiac death was reported more frequent in the PCI group than in the non-PCI group (2.7% vs. 0.06%) [43].

Other authors investigated the impact of PCI for CTO in patients presenting with STEMI on both imagistic (CMR) and clinical outcomes (EXPLORE trial) [44]. In this study, 304 patients were enrolled and randomly divided into two treatment arms: early PCI for the CTO group and medical-only management of the CTO group [44]. No differences were reported between groups regarding LVEF (*p* = 0.60) and left ventricular end-diastolic volume (*p* = 0.70) at the 4-month follow-up. Nevertheless, when CTO was located on the left anterior descending artery, PCI was associated with an increased LVEF compared to conservative treatment (47.2 ± 12.3% vs. 40.4 ± 11.9%; *p* = 0.02). Also, major adverse coronary events were similar in both groups (*p* = 0.25) [44].

A meta-analysis explored the efficacy and safety of staged CTO-PCI versus culprit-only revascularization in patients with STEMI (including the EXPLORE trial) [45]. Culprit-only revascularization was associated with an increased all-cause mortality (OR 2.89, 95% CI, 2.09–4.0), cardiac mortality (OR 3.12, 95% CI, 2.05–4.75), stroke (OR 2.80, 95% CI, 1.04–7.53), and heart failure (OR 1.99, 95% CI, 1.22–3.24). However, the effect exhibited considerable variation between studies, highlighting the need for large randomized clinical trials to confirm these results [45]. Nevertheless, viability assessment was not universal in clinical studies, potentially limiting the generalizability of the findings. The enrolled cohort may have predominantly comprised low-risk CTO procedures, excluding certain techniques. Hence, refined indications likely center around symptoms post-culprit ACS lesion treatment, anatomical considerations, such as proximal LAD involvement or triple-vessel disease with documented viability, and an acceptable risk profile for intervention. These factors underscore the critical importance of tailored patient selection in optimizing outcomes.

## 7. Conclusions

In conclusion, managing multivessel CAD in ACS patients remains a complex and evolving challenge. The existing literature emphasizes the significance of achieving complete myocardial revascularization to optimize short-term and long-term outcomes. Recent evidence from clinical trials, including the 2023 ESC guidelines, supports the complete revascularization strategies, either during the index primary PCI or within a short timeframe following culprit lesion PCI. However, carefully considering individual patient characteristics, coronary stenosis complexity, and clinical context is crucial in decision-making. Furthermore, the timing of interventions, particularly in patients with cardiogenic shock, requires a nuanced approach to balance short-term benefits and long-term outcomes. Moreover, optimal revascularization strategies for CTO in non-culprit arteries require further research through large randomized clinical trials to guide evidence-based practices. Ultimately, refining clinical and interventional strategies for non-culprit lesion management in ACS patients is an ongoing process that demands a comprehensive and individualized approach.

## Figures and Tables

**Table 1:** Key messages on culprit-only vs. complete myocardial revascularization.

(1) Complete revascularization (rather than culprit-only revascularization) is indicated in patients with STEMI and multivessel CAD to reduce the 12-month risk of adverse events in patients who underwent primary PCI.
(2) Staged complete myocardial revascularization is superior to culprit lesion-only PCI for reducing the composite risk of cardiovascular death or myocardial infarction.
(3) Multivessel PCI rather than culprit-only PCI should be considered in NSTEMI patients to reduce the long-term risk of major adverse events and unplanned revascularization.
(4) It is reasonable to perform immediate multivessel PCI in patients with NSTEMI unless they exhibit complex coronary lesions when a staged revascularization strategy should be adopted.
(5) Complete myocardial revascularization is recommended by both ACC/AHA and ESC guidelines.
(6) The decision of complete myocardial revascularization should be individualized, considering coronary stenosis severity, clinical status, and comorbidities.
(7) Complete myocardial revascularization (PCI or CABG) is a cornerstone of ACS management to achieve optimal patient outcomes.
ACS = acute coronary syndrome; CABG = coronary artery bypass grafting; CAD = coronary artery disease; NSTEMI = non-ST-elevation myocardial infarction; PCI = percutaneous coronary intervention; STEMI = ST-elevation myocardial infarction.

**Table 2:** Key messages on immediate versus staged complete revascularization.

(1) Immediate multivessel PCI should be considered to improve long-term outcomes in patients with ACS and multivessel CAD (all amendable to PCI).
(2) The decision of whether to undergo immediate or staged non-culprit PCI should be made on a case-by-case basis, considering clinical status and the severity of CAD.
(3) Immediate multivessel PCI could be considered in hemodynamically stable STEMI patients without left main disease who do not require emergency cardiac surgery (MULTISTARS AMI trial).
(4) It is reasonable to perform immediate multivessel PCI in patients with NSTEMI unless they exhibit complex coronary lesions when a staged revascularization strategy should be adopted.
(5) Further research is needed to clarify the long-term benefits (beyond one year) and risks of immediate versus staged complete revascularization of non-culprit lesions in ACS patients.
(6) The severity of non-culprit coronary stenosis might be overestimated during primary PCI, leading to unnecessary PCI and stenting (small observational data).
ACS = acute coronary syndrome; CAD = coronary artery disease; PCI = percutaneous coronary intervention.

**Table 3:** Key messages on myocardial revascularization in cardiogenic shock.

(1) Culprit-only PCI (rather than multivessel PCI) is recommended as the initial treatment strategy to reduce short-term mortality in ACS patients with cardiogenic shock (based on the CULPRIT-SHOCK trial).
(2) Complete myocardial revascularization should be achieved early after primary PCI to improve long-term outcomes (staged PCI).
(3) Long-term outcomes after culprit-only PCI include a higher risk of repeated revascularization and rehospitalization for heart failure.
(4) The choice between PCI and CABG for revascularization of non-culprit lesions depends on individual patient factors (complexity of multivessel CAD, failed primary PCI, incomplete revascularization, and mechanical complications).
ACS = acute coronary syndrome; CABG = coronary artery bypass grafting; CAD = coronary artery disease; PCI = percutaneous coronary intervention.

**Table 4:** Key messages on optimal revascularization strategy (PCI vs. CABG) for non-culprit coronary stenosis.

(1) Choosing the optimal non-culprit coronary stenosis revascularization strategy (PCI vs. CABG) for ACS patients requires careful consideration of individual characteristics, disease severity, and potential benefits and risks of each option.
(2) CABG may be the preferred option in specific scenarios, including failed PCI, incomplete revascularization, and mechanical complications.
(3) PCI for multivessel CAD could increase the risk of unplanned revascularization compared to CABG.
(4) CABG is associated with an increased risk of myocardial infarction, stroke, acute renal injury, and bleeding as compared to PCI.
(5) The timing of CABG surgery for non-culprit stenosis plays a crucial role in patient outcomes. Postponing CABG for non-culprit coronary stenosis can significantly reduce the risk of in-hospital mortality (in patients not requiring immediate cardiac surgery).
(6) Large randomized clinical trials are required to explore non-culprit coronary stenosis revascularization by CABG vs. PCI in patients with ACS (including the optimal intervention timing).
ACS = acute coronary syndrome; CABG = coronary artery bypass grafting; CAD = coronary artery disease; PCI = percutaneous coronary intervention.

**Table 5:** Key messages related to CTO revascularization in ACS patients.

(1) The coexistence of CTO in non-culprit coronary arteries negatively impacts the outcomes of patients with myocardial infarction.
(2) Complete myocardial revascularization is the recommended goal for ACS management, but the optimal approach for CTO patients with myocardial infarction remains unclear.
(3) PCI may be a beneficial treatment option for non-culprit CTOs in patients with ACS. However, the decision to proceed should be made on a case-by-case basis, considering the patient’s clinical profile, CTO characteristics, and local interventional expertise.
(4) PCI, CABG, and hybrid revascularization strategies can be considered in the context of ACS based on patient-specific factors and lesion complexity.
(5) Large randomized clinical trials are required to confirm the benefit of myocardial revascularization in ACS patients with non-culprit CTO.
ACS = acute coronary syndrome; CABG = coronary artery bypass grafting; CTO = chronic total occlusion; PCI = percutaneous coronary intervention.

## Data Availability

Not applicable.
